# O uso inconsistente de preservativo com parceiros regulares e casuais entre homens que fazem sexo com homens no Brasil: um estudo do tipo *respondent-driven sampling*

**DOI:** 10.1590/0102-311XPT113825

**Published:** 2026-10-12

**Authors:** Jonatan da Rosa Pereira da Silva, Flávia Bulegon Pilecco, Andréa Fachel Leal, Laio Magno, Ines Dourado, Maria Amélia Sousa Mascena Veras, Ligia Regina Sansigolo Kerr, Daniela Riva Knauth

**Affiliations:** 1 Universidade Federal do Rio Grande do Sul, Porto Alegre, Brasil.; 2 Universidade Federal de Minas Gerais, Belo Horizonte, Brasil.; 3 Universidade do Estado da Bahia, Salvador, Brasil.; 4 Universidade Federal da Bahia, Salvador, Brasil.; 5 Faculdade de Ciências Médicas da Santa Casa de São Paulo, São Paulo, Brasil.; 6 Universidade Federal do Ceará, Fortaleza, Brasil.

**Keywords:** Homens que Fazem Sexo com Homens, HIV, Síndrome da Imunodeficiência Adquirida, Parceiros Sexuais, Preservativos, Men Who Have Sex With Men, HIV, Acquired Immunodeficiency Syndrome, Sexual Partners, Condoms, Hombres que Hacen Sexo con Hombres, VIH, Síndrome de Inmunodeficiencia Adquirida, Parejas Sexuales, Condones

## Abstract

O objetivo foi mensurar os fatores associados ao uso inconsistente de preservativo com parceiros regulares e casuais entre homens que fazem sexo com homens (HSH) no Brasil. Foi realizado um estudo transversal, com HSH adultos, recrutados por meio da técnica *respondent-driven sampling*, em 12 capitais brasileiras, em 2016. O uso inconsistente de preservativo foi mensurado por meio das relações sexuais anais insertivas e receptivas ocorridas nos últimos seis meses e na última relação sexual. A associação entre as variáveis independentes e o uso inconsistente de preservativo foi mensurada usando-se a regressão de Poisson com variância robusta, com estimação de razões de prevalência ajustadas e intervalos de 95% de confiança. Foram analisados dados de 1.782 HSH. Quase 75% deles fizeram uso inconsistente de preservativo (46,2% com parceiros regulares e casuais, 23,1% apenas com parceiros regulares e 8,8% apenas com parceiros casuais). Em todas as análises, a prevalência de uso inconsistente de preservativo foi maior entre HSH mais jovens (< 25 anos), entre os que afirmaram aceitar fazer sexo sem usar preservativo quando confiavam no parceiro sexual e os que não fizeram uso de preservativo na primeira relação sexual. Ações em saúde para a promoção do uso de preservativo entre HSH devem considerar aspectos geracionais e comportamentais. O uso de preservativo deve ser incentivado entre HSH mais jovens e em relações em que existe confiança entre os parceiros, pois essas relações também carregam risco de infecção. Ainda, o uso de preservativo precisa ser promovido em conjunto com outras estratégias, promovendo a prevenção combinada ao HIV.

## Abstract (en)

We aimed to analyze the factors associated with inconsistent condom use among men who have sex with men (MSM) in Brazil. This cross-sectional study was carried out with adult MSM, recruited via respondent-driven sampling in 12 Brazilian capitals in 2016. Inconsistent condom use was measured by insertive and receptive anal intercourse in the previous six months and in the last sexual intercourse. The association between the independent variables and inconsistent condom use was measured using Poisson regression with robust variance and the estimation of adjusted prevalence ratios and 95% confidence intervals. Almost 75% of participants showed inconsistent condom use (46.2% with regular and casual partners, 23.1% only with regular partners, and 8.8% only with casual partners). In all analyses, inconsistent condom use prevalence was higher in younger MSM (< 25 years), in those who accept to have sex without condoms and who trusted their partners, and in those that used no condoms in their first sexual relation. Health actions to promote condom use in MSM should consider generational and behavioral aspects. Condom use should be encouraged in younger MSM and in relationships with trusting partners as these relationships also carry infection risk. The use of condoms must be fostered with other strategies, promoting combined HIV prevention.

## Resumen

El objetivo fue medir los factores asociados con el uso inconsistente del condón con parejas habituales y ocasionales entre hombres que tienen sexo con hombres (HSH) en Brasil. En el 2016 se realizó un estudio transversal con HSH adultos, reclutados mediante la técnica *respondent-driven sampling*, en 12 capitales brasileñas. El uso inconsistente del condón se midió mediante las relaciones sexuales anales insertivas y receptivas que tuvieron lugar en los últimos seis meses y durante la última relación sexual. La asociación entre las variables independientes y el uso inconsistente del condón se midió utilizando la regresión de Poisson con varianza robusta, con estimación de razones de prevalencia ajustadas e intervalos de 95% de confianza. Se analizaron datos de 1.782 HSH. Casi el 75% de ellos hizo un uso inconsistente del preservativo (el 46,2% con parejas habituales y ocasionales, el 23,1% solo con parejas habituales y el 8,8% solo con parejas ocasionales). En todos los análisis, la prevalencia de uso inconsistente del condón fue mayor entre HSH más jóvenes (< 25 años), entre aquellos que dijeron que aceptaban hacer sexo sin preservativo cuando confiaban en su pareja sexual y entre aquellos que no usaron preservativo durante su primera relación sexual. Las iniciativas de salud para promover el uso del preservativo entre HSH deben tener en cuenta los aspectos generacionales y conductuales. Se debe fomentar el uso del preservativo entre HSH más jóvenes y en relaciones en las que existe confianza entre los miembros de la pareja, ya que estas relaciones también conllevan un riesgo de infección. Además, es necesario promover el uso del preservativo junto con otras estrategias que fomenten la prevención combinada del VIH.

## Introdução

Mesmo após mais de quatro décadas desde o surgimento dos primeiros casos de aids, homens que fazem sexo com homens (HSH) permanecem entre os grupos mais afetados pela infecção ao HIV ^1^. O crescimento do número de casos de HIV entre HSH tem sido documentado em diferentes regiões do mundo. Em 2010, esses homens corresponderam a 11% das novas infecções por HIV entre indivíduos de 15-49 anos. Em 2022, corresponderam a 20% das novas infecções ^2^.

Assim como no cenário internacional, no Brasil a população de HSH também está mais vulnerável à infecção pelo HIV. Em 2010, enquanto a prevalência de HIV na população em geral era de cerca de 0,6%, entre HSH a prevalência estimada foi de 10,5%. Em 2016, a prevalência para a população em geral foi de 0,4% e para a população HSH 18,4%. Embora a carência de novas estimativas nacionais para HSH, dados do Ministério da Saúde indicam que homens permanecem sendo mais afetados pela epidemia de HIV no país, pois correspondem à maioria dos casos notificados entre 1991 e setembro de 2025 (65,9%, 450.922 casos) ^3^ ^,^ ^4^ ^,^ ^5^.

O cenário epidemiológico apresentado torna evidente a necessidade da combinação de diferentes estratégias de prevenção ao HIV para a população HSH. Nesse contexto, a introdução da prevenção combinada ao HIV no âmbito do Sistema Único de Saúde (SUS), em 2017, trouxe importantes avanços para o enfrentamento da epidemia entre essa população mediante a disponibilização de diferentes tecnologias biomédicas (profilaxia pré-exposição - PrEP, profilaxia pós-exposição - PeP, testagem para HIV e outras infecções sexualmente transmissíveis - IST) aliadas a intervenções comportamentais e estruturais ^6^ ^,^ ^7^ ^,^ ^8^.

Entretanto, a disponibilização de novas tecnologias não garante o seu acesso para a população, especialmente para indivíduos sujeitos a maiores vulnerabilidades que podem encontrar mais dificuldades de acesso aos serviços de saúde ^9^ ^,^ ^10^. Conforme dados do Painel PrEP ^11^, do Ministério da Saúde, até o momento a PrEP permanece sendo mais utilizada por HSH mais jovens, brancos e mais escolarizados. Essas evidências ressaltam que estratégias de prevenção mais acessíveis, como o preservativo ^7^, permanecem sendo importantes para a prevenção do HIV entre a população HSH brasileira.

O uso de preservativo entre a população HSH foi estudado majoritariamente na literatura internacional. Os resultados indicam prevalências de uso inconsistente de preservativo com parceiros casuais que variam entre 43,9%, no Chile ^12^, 52%, na Suíça ^13^, e 37% ^14^ e 48,5% ^15^ com parceiros regulares, no Japão e em Hong Kong (China), respectivamente. Esses estudos indicam que variáveis sociodemográficas (idade, raça/cor de pele, escolaridade, renda) ^16^ ^,^ ^17^, relacionadas ao comportamento sexual (número de parceiros sexuais, histórico de IST, prática sexual insertiva ou receptiva) ^12^ ^,^ ^18^ ^,^ ^19^ ^,^ ^20^ e estruturais (como a participação em atividades de apoio social em organizações não governamentais - ONG - e o acesso aos serviços de saúde) estão associadas ao uso inconsistente de preservativo entre HSH ^6^ ^,^ ^10^ ^,^ ^18^.

No Brasil, o uso inconsistente de preservativo entre HSH ainda é uma temática pouco explorada ^6^. Além disso, os estudos disponíveis investigaram relações com parceiros casuais ^21^, comerciais ^18^ ou em relações anais receptivas ^19^ ^,^ ^22^, tornando o uso inconsistente de preservativo com parceiros regulares e o uso inconsistente de preservativo com mais de um tipo de parceiro uma lacuna ainda a ser explorada.

Frente ao exposto, este estudo teve como objetivo mensurar os fatores associados ao uso inconsistente de preservativo com parceiros regulares e casuais entre HSH no Brasil.

## Métodos

Os dados analisados são provenientes da *Pesquisa Nacional de Vigilância Biológica e Comportamental do HIV, Sífilis, Hepatites B e C entre HSH no Brasil* ^23^. Um estudo transversal, com 4.176 HSH, conduzido entre junho e dezembro de 2016, em 12 capitais brasileiras: Manaus e Belém (Região Norte); Fortaleza, Recife e Salvador (Região Nordeste); Brasília e Campo Grande (Região Centro-oeste); Belo Horizonte, Rio de Janeiro e São Paulo (Região Sudeste) e Curitiba e Porto Alegre (Região Sul).

Participaram do estudo homens cisgêneros, com no mínimo 18 anos, que praticaram sexo oral ou anal com outro homem nos 12 meses que antecederam a coleta de dados e que viviam, trabalhavam ou estudavam em uma das cidades onde o estudo estava sendo desenvolvido.

A técnica *respondent-driven sampling* (RDS) foi utilizada para o recrutamento dos participantes. Antes do recrutamento, uma pesquisa formativa foi conduzida por meio de entrevistas em profundidade e grupos focais, para conhecer os aspectos organizacionais, geográficos e das redes de relacionamentos dos HSH de cada cidade. Entre cinco e seis HSH foram selecionados em cada cidade para iniciar o recrutamento. Esses participantes, denominados sementes, foram escolhidos com base em suas redes de relacionamentos e características sociodemográficas, com vistas a obter uma amostra diversificada de HSH. Cada um recebeu três cupons para convidar outros homens de sua rede para participar da pesquisa. Esse processo foi repetido sucessivamente até que o tamanho amostral previamente estipulado para cada cidade fosse alcançado. Cada participante recebeu um ressarcimento para cobrir as suas despesas com deslocamento e alimentação para a participação na pesquisa. Além disso, um ressarcimento secundário foi creditado a cada convidado que os participantes recrutavam para o estudo, por meio dos convites recebidos (no máximo três convites por participante).

Os dados foram coletados com o uso de *tablets*. Materiais educativos sobre IST, preservativos e lubrificantes foram ofertados para todos os participantes. Foi disponibilizado aconselhamento com profissional de saúde após a realização dos testes rápidos para HIV, sífilis e hepatites B e C. Todos os participantes assinaram formulários de consentimento para a entrevista e os testes realizados. A pesquisa foi aprovada pelo Comitê de Ética em Pesquisa da Universidade Federal do Ceará (CAAE: 43133915.9.0000.5054, parecer nº 1.024.053) e pelas demais instituições envolvidas no estudo. Mais detalhes sobre a pesquisa podem ser encontrados em outras publicações ^18^ ^,^ ^19^ ^,^ ^21^ ^,^ ^23^ ^,^ ^24^.

Para este estudo foram analisados os dados dos HSH que reportaram relações sexuais com parceiros regulares e com parceiros casuais nos últimos seis meses (n = 1.782). Parceiro regular foi definido como alguém com quem o participante mantinha relações sexuais regularmente, incluindo namorado, ficante, marido ou alguém com quem se sentia afetivamente comprometido. Parceiro casual foi definido como alguém com quem o participante teve relações sexuais sem nenhuma regularidade e que não envolveu a troca de dinheiro ou de bens materiais.

O uso de preservativo entre os HSH foi coletado por meio das relações anais insertivas e/ou receptivas dos últimos seis meses e na última relação sexual. Os HSH que declararam não ter usado preservativo em alguma dessas relações foram categorizados como uso inconsistente de preservativo. Com base nisso, foram analisados três desfechos neste estudo:

(1) Uso inconsistente de preservativo com parceiros regulares e casuais: HSH com uso inconsistente de preservativo com ambos os tipos de parceiros sexuais (n = 797);

(2) Uso inconsistente de preservativo com parceiros regulares: HSH que usaram preservativo com parceiros casuais e tiveram uso inconsistente de preservativo apenas com parceiros regulares (n = 394);

(3) Uso inconsistente de preservativo com parceiros casuais: HSH que usaram preservativo com parceiros regulares e tiveram uso inconsistente de preservativo apenas com parceiros casuais (n = 144).

Os HSH que fizeram uso consistente de preservativo nas relações com parceiros regulares e casuais foram utilizados como grupo de comparação para as análises (n = 394).

As variáveis independentes do estudo foram:

(1) Sociodemográficas e econômicas: idade em anos (< 25, ≥ 25); cor de pele autodeclarada (branca, não branca); escolaridade (≤ Ensino Médio incompleto e ≥ Ensino Médio completo); situação conjugal (sem companheiro [solteiro/viúvo/divorciado] e com companheiro [casado/namorando/morando junto]); classe econômica (A/B [alta], C [média], D/E [baixa]), classificada de acordo com o Critério Brasileiro de Classificação Econômica; identidade sexual (gay/homossexual, bissexual e heterossexual/outros); e confiança no parceiro para fazer sexo sem usar preservativo (sim, não), mensurada por meio da seguinte questão: “Se você sente confiança em seu parceiro, você aceita fazer sexo sem usar preservativo?”;

(2) Comportamento sexual: uso de preservativo na primeira relação sexual (sim, não), número de parceiros sexuais nos últimos seis meses, histórico de IST (sim, não), mensurado pela seguinte questão: “Algum profissional de saúde já te disse que você estava com uma IST?”;

(3) Relacionadas com os serviços de saúde: realização de teste HIV durante a vida (sim, não), mensurada pela questão: “Você já realizou teste para HIV/aids em um serviço de saúde alguma vez na vida?”; e

(4) Participação em atividades de apoio social sobre HIV em ONG (ONG HIV) (sim, não).

Os dados de todas as cidades participantes do estudo foram agrupados em um único banco de dados. A amostra foi, então, ponderada em função do tamanho da rede de relações dos participantes, com base nas estimativas realizadas usando-se o software RDS Analyst versão 1.7-16 (https://hpmrg.org/rdsanalyst/), utilizando o estimador de Gile. Para incorporar a ponderação da amostra, todas as análises foram realizadas dentro do comando *Survey Data Analysis* do software Stata 12.0 (https://www.stata.com).

Estatísticas descritivas foram utilizadas para descrever a amostra de acordo com as variáveis independentes e os desfechos do estudo (Tabelas 1 e 2). Modelos multivariáveis de Poisson com variância robusta foram construídos para estimar as razões de prevalências ajustadas (RPa) e intervalos de 95% de confiança (IC95%), para medir a associação entre as variáveis independentes e os desfechos de uso inconsistente de preservativo (Tabelas 3, 4 e 5). Em todas as análises, o uso consistente de preservativo com parceiros regulares e casuais foi utilizado como grupo de referência. A seleção de variáveis para os modelos foi baseada nas análises não ajustadas (p ≤ 0,20) ^25^ e em sua relevância epidemiológica para o desfecho, considerando estudos prévios sobre o uso inconsistente de preservativo entre HSH ^12^ ^,^ ^13^ ^,^ ^14^ ^,^ ^15^ ^,^ ^17^ ^,^ ^18^. Foram consideradas estatisticamente significativas as variáveis com valor de p < 0,05 no modelo ajustado.

**Tabela 1:** Características sociodemográficas dos homens que fazem sexo com homens.

Variáveis	Uso inconsistente de preservativo [n (%)]			Uso consistente de preservativo [n (%)]
	Com parceiros regulares e casuais	Com parceiros regulares	Com parceiros casuais	
Idade (anos)				
< 25	502 (64,2)	249 (66,3)	96 (75,4)	262 (53,7)
≥ 25	295 (35,8)	145 (33,7)	48 (24,6)	185 (46,3)
Cor de pele				
Branca	254 (34)	143 (42,3)	47 (30,5)	145 (28,6)
Não branca	543 (66)	251 (57,7)	97 (69,5)	302 (71,4)
Escolaridade				
≤ Ensino Médio incompleto	171 (24,4)	60 (15,5)	30 (25,5)	82 (18,7)
≥ Ensino Médio completo	626 (75,6)	334 (84,5)	114 (74,5)	365 (81,3)
Situação conjugal				
Solteiro	685 (87,6)	344 (77,9)	130 (87,8)	405 (87,6)
Com companheiro(a)	112 (12,4)	50 (22,1)	14 (12,2)	42 (12,4)
Classe econômica				
A/B	378 (40,1)	208 (51,2)	68 (43,9)	241 (59,6)
C	326 (49,4)	161 (44,7)	61 (49,9)	167 (29,9)
D/E	93 (10,5)	25 (4,1)	15 (6,2)	39 (10,5)
Identidade sexual				
Heterossexual/Outros	305 (44,1)	123 (30,6)	48 (31,6)	156 (27,3)
Bissexual	100 (10,7)	34 (7,5)	14 (19,5)	60 (13,1)
Gay/Homossexual	392 (45,2)	237 (61,9)	82 (48,9)	231 (59,6)
Aceita fazer sexo sem usar preservativo quando sente confiança no parceiro				
Não	381 (43,7)	229 (58,5)	101 (79,6)	357 (88,4)
Sim	416 (56,3)	165 (41,5)	43 (20,4)	90 (11,6)

Nota: dados apresentados por frequência absoluta (não ponderada) e frequência relativa (ponderada).

**Tabela 2:** Características relativas ao comportamento sexual, realização de teste para HIV durante a vida e participação em atividades de organizações não governamentais (ONG) que trabalham com HIV dos homens que fazem sexo com homens.

Variáveis	Uso inconsistente de preservativo [n (%)]			Uso consistente de preservativo [n (%)]
	Com parceiros regulares e casuais	Com parceiros regulares	Com parceiros casuais	
Uso de preservativo na primeira relação sexual				
Sim	260 (30,4)	164 (51,5)	66 (42,6)	259 (63,9)
Não	537 (69,6)	230 (48,5)	78 (57,4)	188 (36,1)
Número de parceiros sexuais nos últimos seis meses [média (DP)]	8,3 (± 0,55)	7,8 (± 0,57)	11,4 (± 2,41)	9,5 (± 1,36)
Histórico de IST				
Sim	554 (71,7)	270 (66,8)	102 (59,4)	316 (63,2)
Não	243 (28,3)	124 (33,2)	42 (40,6)	131 (36,8)
Já realizou teste para HIV alguma vez na vida				
Sim	571 (68,3)	305 (76)	130 (87,8)	334 (74,1)
Não	226 (31,7)	89 (24)	14 (12,2)	113 (25,9)
Participou de atividades de apoio social em ONG HIV				
Sim	109 (11,2)	52 (11,2)	26 (23,1)	72 (19)
Não	688 (88,8)	342 (88,8)	118 (76,9)	375 (81)

DP: desvio padrão.

**Tabela 3:** Fatores associados ao uso inconsistente de preservativo com parceiros regulares e casuais entre os homens que fazem sexo com homens.

Variáveis	Modelo não ajustado		Modelo ajustado	
	RP bruta (IC95%) *	Valor de p	RP ajustada (IC95%) *	Valor de p **
Idade (anos)				
≥ 25	Referência	< 0,0001	Referência	0.001
< 25	1,18 (1,08-1,28)		1,19 (1,09-1,27)	
Cor de pele				
Branca	Referência	0,314	Referência	0,233
Não branca	0,91 (0,76-1,09)		0,91 (0,79-1,05)	
Escolaridade				
≥ Ensino Médio completo	Referência	0,256	-	-
≤ Ensino Médio incompleto	1,12 (0,91-1,38)		-	-
Situação conjugal				
Sem companheiro(a)	Referência	0,998	-	-
Com companheiro(a)	0,99 (0,72-1,38)		-	-
Classe econômica				
A/B	Referência		Referência	
C	1,38 (1,12-1,70)	0,002	1,29 (0,90-1,51)	0.232
D/E	1,18 (0,90-1,55)	0,223	1,13 (0,91-1,41)	0,247
Identidade sexual				
Gay/Homossexual	Referência		Referência	
Bissexual	1,03 (0,85-1,25)	0,729	0,94 (0,77-1,15)	0,576
Heterossexual	1,30 (1,08-1,57)	0,005	1,13 (0,94-1,35)	0,181
Aceita não usar preservativo se sente confiança no parceiro				
Não	Referência	< 0,0001	Referência	< 0,0001
Sim	1,96 (1,67-2,30)		1,73 (1,46-2,05)	
Uso de preservativo na primeira relação sexual				
Sim	Referência	< 0,0001	Referência	< 0,0001
Não	1,72 (1,40-2,11)		1,50 (1,28-1,75)	
Número de parceiros sexuais nos últimos seis meses	0,99 (0,98-1,00)	0,304	0,99 (0,98-1,00)	0,436
Histórico de IST				
Não	Referência	0,267	-	-
Sim	0,85 (0,65-1,12)		-	-
Já realizou teste para HIV alguma vez na vida				
Sim	Referência	0,112	Referência	0,712
Não	1,10 (0,97-1,25)		0,97 (0,85-1,11)	
Participa de atividades em ONG HIV				
Sim	Referência	0,184	1,17 (0,84-1,61)	0,328
Não	1,30 (0,87-1,94)		1,17 (0,84-1,61)	

IC95%: intervalo de 95% de confiança; IST: infecções sexualmente transmissíveis; ONG HIV: organização não governamental que trabalha com HIV; RP: razão de prevalência.

Nota: valores em negrito indicam estatísticas significativas em nível de p.

* Estimativas obtidas por meio do modelo de regressão de Poisson com variância robusta;

** Valor de p associado ao teste de Wald.

**Tabela 4:** Fatores associados ao uso inconsistente de preservativo apenas com parceiros regulares entre homens que fazem sexo com homens.

Variáveis	Modelo não ajustado		Modelo ajustado	
	RP bruta (IC95%) *	Valor de p	RP ajustada (IC95%) *	Valor de p **
Idade (anos)				
≥ 25	Referência	0,018	Referência	< 0,0001
< 25	1,33 (1,05-1,67)		1,47 (1,23-1,76)	
Cor de pele				
Branca	Referência	0,134	Referência	0.155
Não branca	0,73 (0,49-1,10)		0,75 (0,58-1,10)	
Escolaridade				
≥ Ensino Médio completo	Referência	0,572	-	-
≤ Ensino Médio incompleto	0,88 (0,57-1,36)		-	-
Situação conjugal				
Sem companheiro(a)	Referência	0,225	-	-
Com companheiro(a)	1,38 (0,81-2,36)		-	-
Classe econômica				
A/B	Referência		Referência	
C	1,31 (0,89-1,93)	0,165	1,31 (0,96-1,78)	0,085
D/E	0,60 (0,27-1,30)	0,195	0,76 (0,35-1,64)	0,491
Identidade sexual				
Gay/Homossexual	Referência		Referência	
Bissexual	0,70 (0,44-1,13)	0,149	0,67 (0,42-1,06)	0,091
Heterossexual	1,03 (0,69-1,54)	0,846	0,99 (0,69-1,41)	0,956
Aceita não usar preservativo se sente confiança no parceiro				
Não	Referência	< 0,0001	Referência	< 0,0001
Sim	2,03 (1,58-2,61)		1,93 (1,37-2,71)	
Uso de preservativo na primeira relação sexual				
Sim	Referência	0,051	Referência	< 0,0001
Não	1,38 (0,99-1,92)		1,57 (1,24-1,99)	
Número de parceiros sexuais nos últimos seis meses	0,99 (0,98-1,00)	0,389	0,99 (0,99-1,00)	0,295
Histórico de IST				
Não	Referência	0,619	-	-
Sim	0,92 (0,66-1,28)		-	-
Já realizou teste para HIV alguma vez na vida				
Sim	Referência	0,779	-	-
Não	0,94 (0,63-1,40)		-	-
Participa de atividades em ONG HIV				
Sim	Referência	0,168	Referência	0,243
Não	1,43 (0,85-2,39)		1,25 (0,85-1,83)	

IC95%: intervalo de 95% de confiança; IST: infecções sexualmente transmissíveis; ONG HIV: organização não governamental que trabalha com HIV; RP: razão de prevalência.

Nota: valores em negrito indicam estatísticas significativas em nível de p.

* Estimativas obtidas por meio do modelo de regressão de Poisson com variância robusta;

** Valor de p associado ao teste de Wald.

**Tabela 5:** Fatores associados ao uso inconsistente de preservativo apenas com parceiros casuaisentre homens que fazem sexo com homens.

Variáveis	Modelo não ajustado		Modelo ajustado	
	RP bruta (IC95%) *	Valor de p	RP ajustada (IC95%) *	Valor de p **
Idade (anos)				
≥ 25	Referência	0,017	Referência	0,043
< 25	2,11 (1,14-3,87)		1,96 (1,02-3,76)	
Cor de pele				
Branca	Referência	0,826	Referência	0,498
Não branca	0,93 (0,49-1,74)		0,83 (0,50-1,40)	
Escolaridade				
≥ Ensino Médio completo	Referência	0,347	-	-
≤ Ensino Médio incompleto	1,33 (0,72-2,44)		-	-
Situação conjugal				
Sem companheiro(a)	Referência	0,968	-	-
Com companheiro(a)	0,98 (0,49-1,96)		-	-
Classe econômica				
A/B	Referência		Referência	
C	1,80 (0,80-4,03)	0,148	1,65 (0,76-3,55)	0,192
D/E	0,83 (0,35-1,98)	0,678	0,79 (0,27-2,27)	0,663
Identidade sexual				
Gay/Homossexual	Referência		Referência	
Bissexual	1,54 (0,61-3,88)	0,348	Referência	0,983
Heterossexual	1,29 (0,54-3,08)	0,552	1,00 (0,52-1,90)	0,988
Aceita não usar preservativo se sente confiança no parceiro				
Não	Referência	0,096	Referência	0,011
Sim	1,59 (0,91-2,77)		1,48 (1,11-2,44)	
Uso de preservativo na primeira relação sexual				
Sim	Referência	< 0,0001	Referência	< 0,0001
Não	1,89 (1,37-2,60)		2,02 (1,58-2,58)	
Número de parceiros sexuais nos últimos seis meses	1,00 (0,98-1,00)	0,442	1,00 (0,98-1,00)	0,352
Histórico de IST				
Não	Referência	0,316	-	-
Sim	0,71 (0,36-1,39)		-	-
Já realizou teste para HIV alguma vez na vida				
Sim	Referência	0,067	Referência	0,068
Não	1,61 (0,96-2,71)		1,47 (0,97-2,23)	
Participa de atividades em ONG HIV				
Sim	Referência	0,609	-	-
Não	0,83 (0,42-1,66)		-	-

IC95%: intervalo de 95% de confiança; IST: infecções sexualmente transmissíveis; ONG HIV: organização não governamental que trabalha com HIV; RP: razão de prevalência.

Nota: valores em negrito indicam estatísticas significativas em nível de p.

* Estimativas obtidas por meio do modelo de regressão de Poisson com variância robusta;

** Valor de p associado ao teste de Wald.

## Resultados

O uso inconsistente de preservativo com parceiros regulares e casuais foi reportado por 42,6% dos HSH (IC95%: 37,6-47,8, n = 797). Já o uso inconsistente de preservativo com parceiros regulares e o uso inconsistente de preservativo com parceiros casuais foram reportados por 23,1% (IC95%: 19,0-27,8, n = 394) e por 8,8% (IC95%: 6,5-11,6, n = 144) dos HSH, respectivamente. A prevalência de uso consistente de preservativo com parceiros regulares e casuais foi de 25,5% (IC95%: 19,7-32,5, n = 447).

A Tabela 1 apresenta a caracterização sociodemográfica dos HSH de acordo com os desfechos do estudo. Os HSH apresentaram um perfil sociodemográfico semelhante: mais jovens (< 25 anos), não brancos, mais escolarizados (≥ Ensino Médio completo), sem companheiro(a), pertencentes às classes econômicas mais altas (A/B e C) e se identificaram como gays/homossexuais.

Quanto à confiança no parceiro, a maioria dos HSH que fizeram uso inconsistente de preservativo com parceiros regulares e casuais afirmou que aceitavam fazer sexo sem usar preservativo quando confiavam no parceiro sexual (56,3%, n = 416). Por outro lado, entre os HSH com uso inconsistente de preservativo com parceiros regulares, os com uso inconsistente de preservativo com parceiros casuais e os com uso consistente de preservativo, a maioria afirmou não aceitar fazer sexo sem preservativo mesmo quando confiavam no parceiro sexual (58,5%, n = 229; 79,6%, n = 101; 88,4%, n = 357, respectivamente).

No que se refere ao comportamento sexual, a maior parte dos HSH com uso inconsistente de preservativo com parceiros regulares e casuais e os com uso inconsistente de preservativo com parceiros casuais afirmou não ter utilizado preservativo na primeira relação sexual (69,6%, n = 537 e 57,4%, n = 78, respectivamente) (Tabela 2). Quanto ao número de parceiros sexuais, os HSH com uso inconsistente de preservativo com parceiros casuais se relacionaram, em média, com 11,4 parceiros sexuais nos últimos seis meses. Por outro lado, HSH com uso inconsistente de preservativo com parceiros regulares apresentaram a menor média de parceiros nos últimos seis meses (7,8 ± 0,57).

A maioria dos HSH afirmou que não possuía histórico de alguma IST (71,7% uso inconsistente de preservativo com parceiros regulares e casuais, 66,8% uso inconsistente de preservativo com parceiros regulares, 72,9% uso inconsistente de preservativo com parceiros casuais e 63,2% uso consistente com parceiros regulares e casuais). A maior parte dos HSH também afirmou já terem realizado teste para HIV alguma vez na vida (68,3% uso inconsistente de preservativo com parceiros regulares e casuais, 76% uso inconsistente de preservativo com parceiros regulares, 59,4% uso inconsistente de preservativo com parceiros casuais e 74,1% uso consistente com parceiros regulares e casuais). Já a participação em atividades de apoio social em ONG HIV foi pouco frequente entre todos os HSH do estudo.

Quanto às nossas análises multivariáveis (Tabela 3), a prevalência de uso inconsistente de preservativo com parceiros regulares e casuais foi maior entre HSH com < 25 anos (RPa = 1,19, IC95%: 1,09-1,27, p < 0,001), que não usaram preservativo na primeira relação sexual (RPa = 1,73, IC95%: 1,46-2,05, p < 0,0001) e que afirmaram aceitar fazer sexo sem preservativo quando confiavam no parceiro sexual (RPa = 1,50, IC95%: 1,28-1,75, p < 0,0001).

A prevalência de uso inconsistente de preservativo com parceiros regulares também foi maior entre HSH com < 25 anos (RPa = 1,47, IC95%: 1,23-1,76, p < 0,0001), que não usaram preservativo na primeira relação sexual (RPa = 1,57, IC95%: 1,24-1,99, p < 0,0001) e que afirmaram aceitar fazer sexo sem preservativo quando confiavam no parceiro sexual (RPa = 1,93, IC95%: 1,37-2,71, p < 0,0001) (Tabela 4).

No caso do uso inconsistente de preservativo com parceiros casuais as mesmas variáveis apresentaram associação significativa no modelo ajustado (ter < 25 anos, RPa = 1,96, IC95%: 1,02-3,76, p = 0,043, não ter usado preservativo na primeira relação sexual, RPa = 2,02, IC95%: 1,58-2,58, p < 0,0001, e aceitar transar sem usar preservativo quando sente confiança no parceiro sexual, RPa = 1,48, IC95%: 1,11-2,44, p = 0,011) (Tabela 5).

## Discussão

Nossos resultados indicaram que quase 75% dos HSH deste estudo relataram o uso inconsistente de preservativo nas relações sexuais nos últimos seis meses e/ou na última relação sexual (42,6% uso inconsistente de preservativo com parceiros regulares e casuais, 23,1% uso inconsistente de preservativo apenas com parceiros regulares e 8,8% apenas com parceiros casuais). A prevalência de uso inconsistente de preservativo foi maior entre HSH mais jovens (< 25 anos), entre aqueles que afirmaram aceitar fazer sexo sem preservativo quando confiam no parceiro sexual e entre os que não utilizaram preservativo na primeira relação sexual, indicando que este desfecho está associado a aspectos geracionais, individuais e relacionados ao comportamento sexual.

Comparações entre estudos sobre uso inconsistente de preservativo entre HSH devem ser realizadas com cautela, uma vez que a literatura não é uniforme quanto à forma de mensuração do uso de preservativo e à definição do tipo de parceria sexual. Para minimizar esse problema, selecionamos estudos realizados no Brasil ou pesquisas que mensuraram o uso inconsistente de preservativo com parceiros regulares e/ou casuais de forma semelhante às definições adotadas em nosso estudo, permitindo contextualizar adequadamente nossos resultados com a literatura existente.

A prevalência de uso inconsistente de preservativo observada em nosso estudo é superior à encontrada em análises que utilizaram dados da mesma pesquisa nacional, as quais indicaram prevalências de 50,8% de uso inconsistente de preservativo com parceiros casuais ^21^ e de 26% com parceiros comerciais ^18^. Entretanto, esses estudos investigaram o uso inconsistente de preservativo considerando apenas um tipo de parceiro, e a nossa análise contemplou a ocorrência do comportamento com mais de um tipo de parceria.

Da mesma forma, a prevalência de uso inconsistente de preservativo encontrada neste estudo é superior à observada em uma pesquisa brasileira que investigou exclusivamente relações sexuais anais receptivas entre HSH, na qual foram identificadas prevalências de 41,9% entre HSH < 25 anos e de 29,7% entre aqueles com ≥ 25 anos ^19^. Mais recentemente, um estudo que analisou dados de 30.089 homens participantes da *Pesquisa Nacional de Saúde* de 2019 indicou que 43,7% dos homens homo/bissexuais relataram uso inconsistente de preservativo nas relações sexuais nos últimos 12 meses. Quando considerada apenas a última relação sexual, a prevalência foi de 19,5% entre esses homens ^26^.

No contexto internacional, a prevalência encontrada em nosso estudo também é superior à observada em pesquisas realizadas com HSH no Chile (43,9% de uso inconsistente de preservativo com parceiros casuais) ^12^ e na Suíça (52%) ^13^, bem como à prevalência de uso inconsistente de preservativo com parceiros regulares identificada em Hong Kong (48,5%) ^15^ e no Japão (37%) ^14^.

Além de elevada quando comparada às prevalências descritas na literatura, a magnitude do uso inconsistente de preservativo observada neste estudo é particularmente preocupante quando analisada à luz do contexto epidemiológico da infecção pelo HIV entre HSH no Brasil. Estudos nacionais indicaram prevalências de HIV elevadas nessa população (10,5% em 2010 e 18,4% em 2016), e dados do Ministério da Saúde mostram a concentração da epidemia entre homens, que representaram 65,9% dos casos notificados entre 1991 e setembro de 2025 ^3^ ^,^ ^4^ ^,^ ^5^. Além disso, outras estratégias biomédicas de prevenção, como a PrEP, foram implantadas no SUS apenas em 2017, após a coleta de dados deste estudo, e continuam sendo utilizadas predominantemente por HSH socialmente menos vulneráveis, especialmente indivíduos brancos (56%) e com maior escolaridade (76%). Ainda, mesmo em 2026 o número de HSH em uso de PrEP no país é de 119.116 usuários, valor inferior ao estimado pelo Ministério da Saúde para alcançar maior efetividade populacional da estratégia ^11^.

Assim como observado em outras pesquisas realizadas no Brasil ^19^, na Índia ^16^ e na Nova Zelândia ^17^, os HSH mais jovens (< 25 anos) apresentaram maior prevalência de uso inconsistente de preservativo em comparação aos HSH com ≥ 25 anos. Estudos sugerem que a incorporação de novas tecnologias no tratamento e na prevenção do HIV e da aids contribuiu para mudanças nas representações sociais da doença, especialmente entre as gerações mais jovens. Informantes da pesquisa formativa que subsidiou este estudo relataram que homossexuais nascidos em gerações mais recentes, particularmente após a introdução da terapia antirretroviral, tendem a perceber a aids como uma doença crônica, e não mais como uma condição fatal ^27^.

Outros fatores também podem contribuir para explicar o maior uso inconsistente de preservativo entre HSH mais jovens, como a chamada “fadiga do uso”, descrita desde a década de 1990 entre HSH, as novas formas de encontrar parceiros sexuais em contextos mediados por redes sociais e aplicativos de relacionamento, e a insuficiência de estratégias de prevenção ao HIV e a aids decorrente da redução ou desestruturação de programas e intervenções de educação sexual ^28^ ^,^ ^29^.

Por outro lado, dados recentes do Ministério da Saúde indicam que, em 2026, HSH nas faixas etárias de 25-29 anos (22,1%) e 30-39 anos (44,6%) representam a maioria dos usuários de PrEP no país. Parte desses indivíduos encontrava-se na faixa etária de < 25 anos no período de coleta de dados desta pesquisa, o que pode sugerir que a implantação da prevenção combinada no âmbito do SUS tenha contribuído para fortalecer a adoção de estratégias de prevenção nesse grupo. O uso de preservativo também pode ter sido reforçado, uma vez que constitui uma das estratégias recomendadas no conjunto de intervenções da prevenção combinada ^11^.

A confiança no parceiro sexual mostrou-se um importante determinante do uso inconsistente de preservativo entre os HSH deste estudo. Na literatura, a confiança é descrita de diferentes maneiras: como um sentimento construído ao longo do relacionamento, como algo que “simplesmente existe” independentemente da duração da relação ou ainda como resultado de acordos explícitos de monogamia, frequentemente associados a sentimentos de amor e intimidade. Em todas essas situações, a confiança tem sido associada à maior frequência de uso inconsistente de preservativo entre HSH, pois parceiros considerados confiáveis são percebidos como apresentando menor risco de infecção por alguma IST, o que justificaria a ocorrência de relações sexuais sem preservativo ^30^ ^,^ ^31^.

Por outro lado, o uso inconsistente de preservativo também tem sido descrito como uma prática utilizada para construir ou demonstrar confiança no relacionamento. Em alguns estudos, HSH relataram que fazer sexo sem preservativo pode ser interpretado como um sinal de envolvimento, comprometimento, amor e monogamia com o parceiro. Nesses casos, a confiança também pode dificultar a negociação do uso de preservativo, uma vez que introduzir esse tema após relações previamente desprotegidas pode gerar suspeitas de traição ou de infidelidade ^30^ ^,^ ^31^.

Em nossas análises, HSH que não utilizaram preservativo na primeira relação sexual apresentaram maior prevalência de uso inconsistente de preservativo. A iniciação sexual tem sido apontada como um importante marcador da saúde sexual ao longo da vida. Estudos indicam que, em comparação com outras populações, HSH tendem a iniciar a vida sexual mais precocemente e com parceiros mais velhos, o que pode dificultar a negociação do uso de preservativo. Além disso, o início mais precoce da vida sexual aumenta o tempo de exposição a comportamentos de risco, uma vez que esses indivíduos podem ter maior número de parceiros ao longo da vida e menor frequência de uso de preservativo ^32^ ^,^ ^33^.

Os resultados deste estudo revelam importantes comportamentos de risco em uma amostra de HSH de 12 cidades brasileiras, contribuindo para a produção de conhecimento sobre o uso inconsistente de preservativo nessa população. Entretanto, algumas limitações devem ser consideradas. Inicialmente, os dados são provenientes de um estudo transversal, no qual não é possível estabelecer relações causais, apenas associações. Além disso, a generalização dos resultados deve ser feita com cautela, uma vez que a amostra foi obtida por meio da RDS. Nossas análises também não consideraram o número de parceiros regulares e casuais relatados pelos participantes, assim, caso um indivíduo tenha tido mais de um parceiro regular ou casual, o uso inconsistente de preservativo pode ter ocorrido apenas com um deles.

Adicionalmente, por se tratar de um comportamento autorreferido, não se pode descartar a possibilidade de viés de informação. Para minimizar esse problema, combinamos duas medidas de uso de preservativo para analisar esse desfecho: o uso em relações sexuais insertivas e/ou receptivas nos últimos seis meses, por ser mais representativo do comportamento sexual, e o uso de preservativo na última relação sexual, medida menos sujeita a viés de memória.

Por fim, os resultados deste estudo indicam que ações de saúde voltadas à redução da infecção pelo HIV entre HSH no Brasil devem priorizar especialmente os indivíduos mais jovens, reforçando a importância do uso consistente de preservativo aliado a outras estratégias biomédicas e comportamentais de prevenção. Além disso, o uso consistente de preservativo deve ser incentivado mesmo em relações marcadas pela confiança entre os parceiros, uma vez que a confiança não elimina o risco de exposição às infecções sexualmente transmissíveis. Estratégias de prevenção que abordem o início da vida sexual também são fundamentais, considerando que HSH que não utilizam preservativo nas primeiras experiências sexuais tendem a manter esse padrão ao longo da vida sexual.

## Data Availability

Os dados de pesquisa estão disponíveis mediante solicitação ao autor de correspondência.
